# Asexual Propagation of Sea Anemones That Host Anemonefishes: Implications for the Marine Ornamental Aquarium Trade and Restocking Programs

**DOI:** 10.1371/journal.pone.0109566

**Published:** 2014-10-14

**Authors:** Anna Scott, Jannah M. Hardefeldt, Karina C. Hall

**Affiliations:** National Marine Science Centre and Marine Ecology Research Centre, School of Environment, Science and Engineering, Southern Cross University, Coffs Harbour, New South Wales, Australia; Zhejiang University, China

## Abstract

Anemonefishes and their host sea anemones form an iconic symbiotic association in reef environments, and are highly sought after in the marine aquarium trade. This study examines asexual propagation as a method for culturing a geographically widespread and commonly traded species of host sea anemone, *Entacmaea quadricolor*. Two experiments were done: the first to establish whether size or colour morph influenced survival after cutting into halves or quarters; and the second to see whether feeding was needed to maximise survival and growth after cutting. Survival rates were high in both experiments, with 89.3 and 93.8% of the anemones cut in half, and 62.5 and 80.4% cut in quarters surviving in experiments 1 and 2, respectively. Anemones that were cut in half were larger in size, and healed and grew quicker than those cut in quarters. However, even though survival was lower when the individuals were cut in quarters, this treatment produced the greatest number of anemones. Feeding increased oral disc diameter growth and reduced wet weight loss, but did not significantly influence pedal disc diameter. Given that the anemones took up to 56 d to form an off-centre mouth, it is highly likely that feeding may have produced greater effect if the experiment was run for longer. This low technology method of propagation could be used to produce individuals throughout the year and the anemones could then be used to supply the aquarium trade or restock depleted habitats, thus supporting biodiversity conservation in coral reef areas.

## Introduction

The trade of marine ornamentals for aquariums is rapidly expanding, causing concerns about the sustainability and environmental impacts of the industry [1]–[3]. Collection often occurs on coral reefs in developing island nations, where it can be difficult to implement effective management [2], [4], [5]. The fishery is highly selective, and harvesting from limited areas has led to localised depletions or extinctions of target species in some cases [6]–[8].

Sea anemones that host symbiotic anemonefishes are highly sought after by aquarists [2], [9]. An alteration in the population dynamics of either partner generally effects the other due to the obligate nature of the symbiosis [6], [8], [10]. These host anemones represent high-value species for collectors (e.g. in the Philippines the price paid to fisherman for anemones can be up to 13 times the amount for anemonefishes), which means they are often preferentially harvested [8]. The development of reliable and cost-effective methods for culturing anemones could facilitate the supply of animals for the aquarium trade, or the restocking of reefs that have already been impacted by natural or anthropogenic disturbances [11]. In doing so, it would create a new and ecologically sustainable industry, and thus support biodiversity conservation in coral reef areas [12], [13].

Anemones could be cultured using sexual reproduction, which has been studied in two (*Entacmaea quadricolor* and *Heteractis crispa*) of the ten species of sea anemones that host anemonefishes [14]–[17]. Both of these anemones have separate sexes, and broadcast spawn gametes for external fertilization and development during predictable annual spawning periods [14], [15], [17], [18]. The larvae settle onto a variety of surfaces, where metamorphosis and development occurs [16]. Alternatively, it may be possible to propagate anemones using asexual reproduction. *Entacmaea quadricolor* and *Heteractis magnifica* can reproduce asexually using longitudinal fission [19], where the anemone divides by stretching in opposite directions, thinning the tissue and tearing perpendicular to the axis of stretch [20], [21]. Porat and Chadwick-Furman [22] cut six *E. quadricolor* in half to generate individuals for a split-pair laboratory experiment with anemonefishes, and found that the majority survived (67%). Likewise, anecdotal evidence from aquarium hobbyists suggests that a variety of anemone species might be able to be propagated by cutting them in half.

This study aimed to determine whether the most geographically widespread host anemone, *E. quadricolor*, could be cultured using fragmentation. We investigated: 1) whether anemones could survive being cut in halves or quarters, and which cut type would produce the maximum number of anemones; 2) if survival was influenced by initial size or colour morph; and 3) whether feeding was needed to maximise survival and growth. Given that this species naturally undergoes longitudinal fission [19], and there is some published [22] and anecdotal evidence to suggest these anemones can survive being fragmented, it was hypothesized that: anemones would survive being cut; that survival would be influenced by size (with larger individuals faring better than smaller individuals) but not colour morph; and that feeding would increase survival and accelerate growth.

## Materials and Methods

### Ethics statement

This work was done under the conditions specified by NSW Fisheries Permit P02/0025-4.0.

### Experiment 1 – survival

Twenty-four *E. quadricolor* were collected on SCUBA from 15–18 m depth on 14 March 2012 at North Solitary Island, Solitary Islands Marine Park, New South Wales, Australia (29°55′S, 153°23′E). Anemones were gently removed from the substratum by hand, placed into a mesh bag, and transferred into a 70-L white polyvinyl chloride (PVC) tub on the boat. They were transported to the National Marine Science Centre, Coffs Harbour and maintained in a shaded rectangular 3 000-L outdoor tank with flow-through (10 L min^−1^) coarse-sand filtered (∼30 µm) seawater for 12 d before the start of the experiment. Seawater was sourced from Charlesworth Bay (30°15′S, 153°8′E) through a gravel filter-box system that was located 150 m offshore and at a depth of approximately 3 m. Half of the anemones had a purple column and brown tentacles with white tips (morph 1); while the other half had a red column and brown tentacles with a white ring below green tips (morph 2). It is likely that morph 1 was male, and morph 2 was female, as Scott and Harrison [17] found that the colour of the column and tentacles generally corresponded to a particular sex at the collection location.

The experiment was done in two 3 000 L tanks that were located outdoors to provide natural photoperiod and light. Shade cloth covers were placed over the tanks to ensure that light induced bleaching did not occur (light levels did not exceed 400 µmol photons m^−2^ s^−1^). Twenty-four white PVC tubs (40 L, 29.5×39 cm wide, 30 cm deep) were evenly distributed between the tanks. Each tub had four circular outlets (2.5 cm diameter) located 3.5 cm below the top that were covered with 1 mm square mesh; a rock (approximately 15×10×6 cm) for habitat; and was supplied with flow-through seawater (sourced as described above but supplied at 0.5 L min^−1^) to maintain ambient seawater temperature (20.5–24.5°C). Temperature was logged every 15 min in each of the tanks using a Thermochron iButton temperature logger (Maxim, USA).

The anemones were weighed on a top-pan balance after gentle squeezing and blotting with absorbent paper to remove as much water as possible (wet weight 61–274 g). Anemones were then randomly allocated to tubs and acclimatised for 3 d before the start of the experiment. Eight individuals (four of each colour morph) were randomly assigned to each of the following treatments: control (uncut), cut in half, or cut in quarters.

At the start of the experiment, the oral disc diameter (ODD) of each anemone was measured along the long and short axes to the nearest mm with vernier callipers and averaged (ODD 109–235 mm). Anemones were then removed from the tubs and fragmented with a 165 mm steel chefs knife. This was done on a plastic cutting board, whilst wearing latex gloves. Tentacles were moved toward the outer edge of the oral disc, and cuts were made through the anemones mouth (along a random plane to the directional mouth). The resulting fragments were immediately placed back into their respective tubs, with the pedal disc directed downwards. The knife and cutting board were cleaned between cutting each anemone. Control anemones were also removed from the tubs; however they were simply placed back into the tubs without cutting. If the water in the tubs became cloudy after cutting a ∼80% water exchange was done to rapidly clear the water.

The number of alive fragments (i.e. survival) was recorded at 6 h, daily for the first week, and then on day 9, 11, 14, 21, 28, and 35. Anemones were fed small pieces of prawn on a weekly basis, starting 7 d after cutting. Tubs were cleaned every 3 d to remove boluses or mucus produced by the anemones and residual food, and to prevent algal build up on the tubs. This was done using a scouring pad and siphon hose.

### Experiment 2 – survival, effect of feeding on size, and visual observations of recovery

The methodology for experiment 2 was similar to that described above, except for the following departures. Forty-two *E. quadricolor* of four colour morphs: i) red column, brown tentacles with white ring below green tips (n = 18); ii) purple column, brown tentacles with white tips (n = 13); iii) light brown column, brown tentacles white ring below brown tips (n = 7); and iv) pink column, brown tentacles with white tips (n = 4), were collected from 12–18 m depth on the 5 October 2012. The anemones were measured before cutting (ODD 98–215 mm, pedal disc diameter [PDD, which was measured using the same methods described for ODD] 45–108 mm, and wet weight 32–171 g). The linear measurements of ODD and PDD were correlated (correlation coefficient r = 0.77), but each only showed weak correlation with wet weight (r = 0.4 for both).

Anemones were randomly allocated directly into 42 separate tubs rather than held temporarily in a holding tank. The tubs were located in three 1 200 L tanks supplied with flow-through seawater (sourced the same way as described in experiment 1, but then filtered to 10 µm with Filtaflo sediment filters and supplied at 0.6 L min-1), which was heated in two 3 000 L header tanks using Aquahort heater/chiller units to maintain a similar temperature range (21–24°C) to that in experiment 1. Temperature was logged every 3 h in twelve tubs (four in each tank).

Fourteen individuals were randomly assigned to each of the treatments: control (no cut), cut in half, or cut in quarters. Half of the anemones from each of these treatments were fed weekly. This feeding frequency was selected as the growth rates of juvenile *Heteractis cripsa* fed once or three-times weekly have been shown to be equivalent [11]. Pieces of prawn flesh were placed onto the oral disc until each anemone was satiated, and consequently the amount of food received by each individual was proportional to its size. The remaining anemones were not provided with any additional heterotrophic food. Although individuals were randomly allocated to the various treatments, the treatment types were divided among the three experimental tanks.

The number of alive fragments (i.e. survival) and their recovery (cut healing status, and whether or not their oral disc was expanded and their pedal disc was attached to the tub) were recorded at 6 h, daily for the first week, and then on day 9, 11, 14, 21, 28, 35, 42, 56, 70, 84 and 105. Throughout the experiment ODD, PDD, and wet weight were used as indicators of anemone size. Measurements of ODD and PDD were first taken one week after cutting to allow the anemones time to re-expand, and were repeated on day 28, 42, 56, 70, 84 and 105. Fragments were weighed immediately after cutting, and then on d 28, 56, 84, and 105. Tubs were cleaned every 2–3 d.

### Data analysis

#### Survival – experiments 1 and 2

Survival differences among the different treatments were investigated using the Fisher's exact test. Variations in survival over time among the different treatments were analysed for each experiment using nonparametric Kaplan-Meier (K-M) product-limit analyses. The census times at which fragments were last observed alive were used as the time of death, so estimates of survival times are slightly conservative. As non-parametric survival analyses cannot explicitly incorporate information from replication, survival curves were estimated for the total number of fragments in each treatment combination pooled across replicate tubs.

Data from experiment 1, were further analysed with a parametric survival analysis (Cox proportional hazards model), to determine whether pre-fragmented size or morph had a significant effect on survival over time. Pre-fragmented sizes were categorised as either small (<100 g) and large (>100 g) for analyses. Survival data were analysed using the ‘survival’ library in R (version 2.11.1, http://www.r-project.org).

#### Growth – experiment 2

After cutting, there were uneven numbers of anemones in each tub (one control, two halves, or four quarters). In tubs containing multiple fragments it was not possible to individually identify each fragment between measurements, so growth data (i.e. wet weight, ODD, and PDD) were averaged per tub for all cut anemones and tubs were used as replicate units for each treatment combination. Actual size measurements were used for all statistical analyses rather than percentage change to alleviate complications associated with negative growth and percentages for statistical computations. To account for the influence of initial size on subsequent growth, the first size measurement of each fragment (immediately after cutting for wet weight and on day 7 for ODD and PDD) was used as a covariate in all analyses. All growth variables were log-transformed to meet the assumptions of normality and homoskedacticity.

To assess the effects of cut types (no cut, half, and quarter), feeding (fed and unfed), and tanks (1, 2, and 3) on growth during the experiment, generalized linear models (GLM) were fitted with the ‘lme4’ package in R, with parameters estimated by restricted maximum likelihood (REML). Baseline models included the design/random variables of initial fragment size as a fixed covariate, and tank and days as random factors. Days were nested within tubs to account for the repeated measures experimental design. The treatment factors of cut types, feeding and days, and associated interaction terms were fitted in various combinations of fixed factors along with the design/random variables in separate GLM, and compared by maximum likelihood comparisons of the change in deviance from the baseline model using the Akaike Information Criterion (AIC).

## Results

### Survival

**Figure 1:**
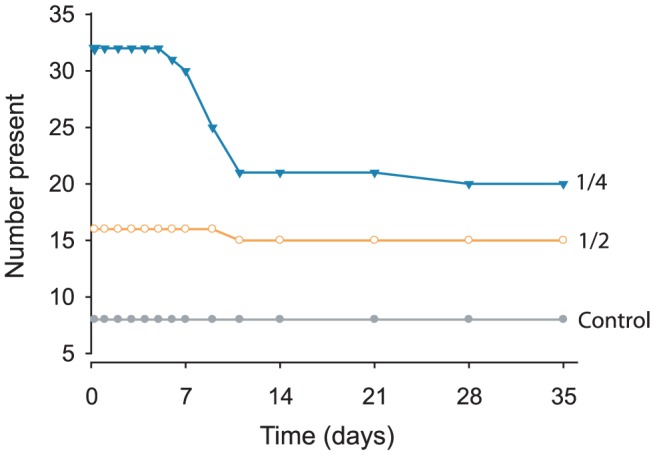
Number of *Entacmaea quadricolor* present in each treatment over time (in days) during experiment 1.

**Figure 2:**
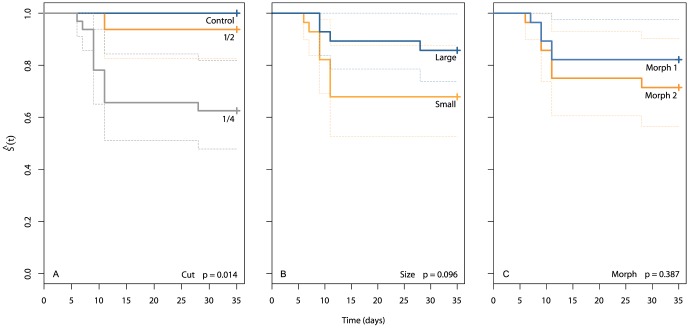
Kaplan-Meier survival [Ŝ(t)] plots for *Entacmaea quadricolor* among different: (a) cut types, (b) sizes, and (c) morphs during experiment 1. Dashed lines represent confidence intervals.

In experiment 1, all of the eight controls survived, along with 15 of the 16 halves (93.8%) (Figure 1). In contrast, significant mortality occurred in the quarters, with 20 of the 32 fragments (62.5%) surviving (Figures 1 and 2a, Fisher's, χ^2^ = 10.6, df = 2, p = 0.005). Nevertheless, anemones cut into quarters still produced the greatest number of anemones at the end of the experiment (20 quarters vs 15 halves). Among the quarters, fragments from morph 1 (75%) had greater survival than those from morph 2 (50%), but this result was not significant (Figure 2b, K-M, Wald  = 1.7, df = 1, p = 0.197). Likewise, although more of the quarters from large anemones survived (75%), compared with those from small anemones (50%), the difference was not significant (Figure 2c, K-M, Wald  = 2.3, df = 1, p = 0.131).

**Figure 3:**
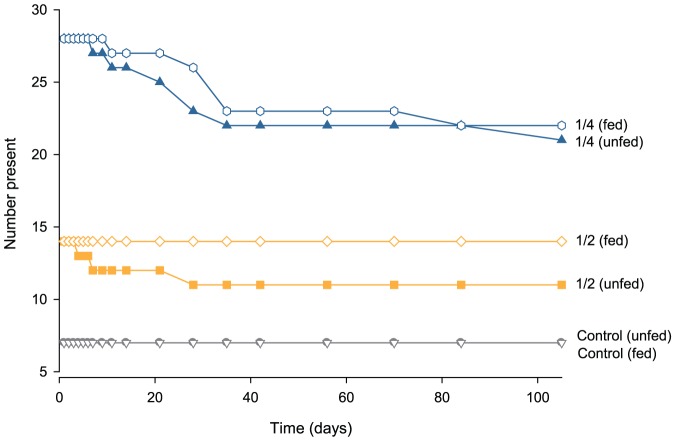
Number of *Entacmaea quadricolor* present in each treatment over time (in days) during experiment 2.

**Figure 4:**
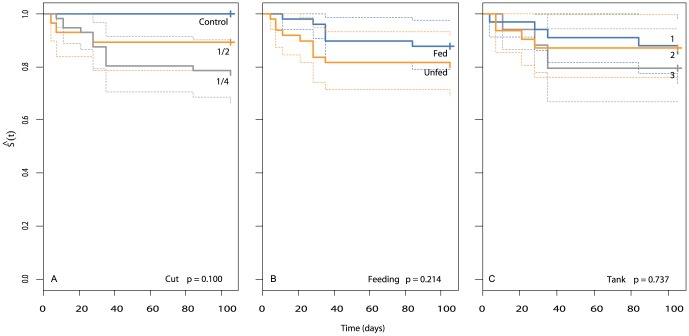
Kaplan-Meier survival [Ŝ(t)] plots for *Entacmaea quadricolor* among different: (a) cut types, (b) feeding treatments, and (c) tanks during experiment 2. Dashed lines represent confidence intervals.

In experiment 2, no significant mortality was recorded among either halves or quarters relative to controls (Figure 3, Fisher's, χ^2^ = 4.2, df = 2, p = 0.124). All controls survived, and 89.3% and 80.4% of halves and quarters survived, respectively. Thus, the anemones cut in quarters produced almost double the number of anemones (n = 45 surviving fragments) than those cut in half (n = 25). Given the low mortality in this experiment, none of factors analysed (cut type, feeding or tanks) had a significant effect on the survival of fragments over time (Figure 4, K-M, p>0.05 in all cases).

### Effects of feeding on size

Larger fragments resulted from cutting larger anemones for a given cut type. This initial fragment size had a significant influence on final fragment size as measured by all three variables, with anemones of a larger initial size remaining larger throughout the experiment (GLM; p<0.001 in all cases).

**Figure 5:**
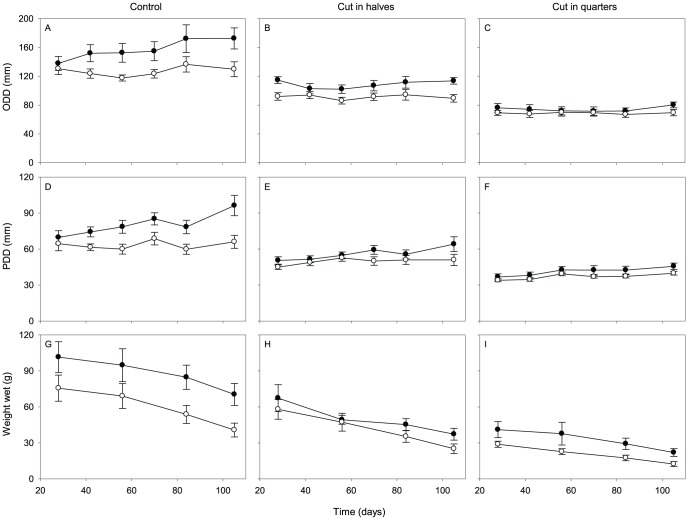
Change in size of *Entacmaea quadricolor* over time (in days) during experiment 2. Estimated means (±SE) of fitted (a–c) ODD, (d–f) PDD, and (g–i) wet weight from generalized linear models.

Although the ODD of the sea anemones showed no consistent positive or negative change over time across all treatments during the experiment (non-significant day effect, GLM, χ^2^ deviance  = 10.0, df = 5, p = 0.08), it did vary significantly among cut types and feeding treatments (Figure 5a–c). The ODD of quarters increased less during the experiment than that of halves or controls (significant cut type effect, GLM, χ^2^ deviance  = 9.9, df = 2, p = 0.007), even after the effect of initial fragment size was accounted for. The ODD of the fed anemones grew larger than unfed anemones (significant feeding effect, GLM, χ^2^ deviance  = 9.5, df = 1, p = 0.002), particularly among controls (Figure 5a) and to a lesser extent among halves later in the experiment (Figure 5b).

In comparison, feeding had no influence on the PDD of the anemones (non-significant feeding effect, GLM, χ^2^ deviance  = 2.6, df = 1, p = 0.108). Nevertheless, PDD generally increased throughout the experiment for all treatment groups (significant day effect, GLM, χ^2^ deviance  = 21.0, df = 5, p<0.001). Cut type also influenced PDD growth, with the controls and halves growing more than the quarters (significant cut type effect, GLM, χ^2^ deviance  = 12.5, df = 2, p = 0.002).

Although all of the anemones gradually decreased in wet weight, including the controls (significant day effect, GLM, χ^2^ deviance  = 68.6, df = 3, p<0.001), those that received food showed reduced wet weight loss in comparison to those that were unfed (significant feeding effect, GLM; χ^2^ deviance  = 5.3, df = 1, p = 0.022). There was an interaction between these two factors, with feeding having a greater influence towards the end of the experiment than at the start (significant feeding × day effect, GLM, χ^2^ deviance  = 9.5, df = 3, p = 0.023).

### Visual observations

**Figure 6:**
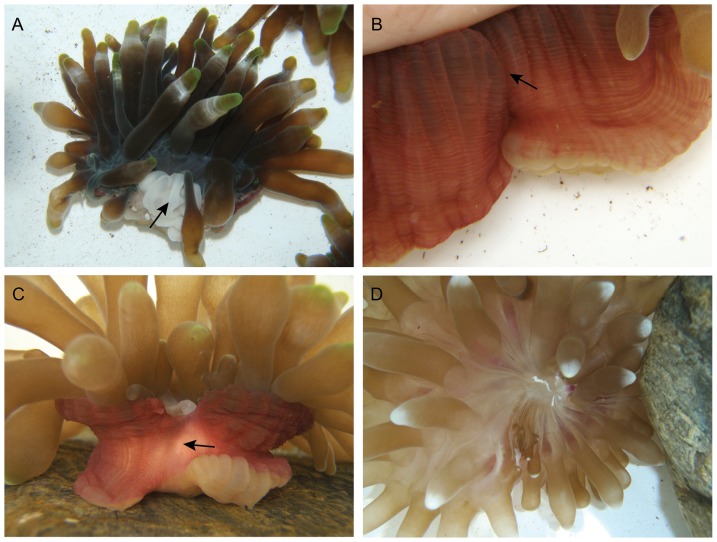
Recovery of *Entacmaea quadricolor* fragments after cutting: (a) open cut, (b) column tissue covering the cut, (c) column coloured scar tissue, and (d) off-centre mouth.

The majority of individuals in all of the treatments had attached to the bottom of the tubs 6 h after the start of experiment 2 (93% of the controls, 96% of the halves, and 89% of the quarters). Likewise, most of the anemones that weren't cut or were cut in half had re-expanded their oral disc and tentacles (93% and 73%, respectively); however only 32% of the quarters had re-expanded. Most anemones still had an open cut at this time (Figure 6a). While in others, the column had started to curl inwards towards the region of the cut (36% of the anemones cut in halves and 7% of those cut in quarters), or the column had completely covered the cut (39% of the anemones cut in half, Figure 6b). After 28 d, scar tissue covered the region where the cut was made in 96% of the cut individuals (Figure 6c).

All of the anemones accepted food 7 d after the start of the experiment (anemones that had been fragmented moved the food towards the area of the cut). By day 56, off-centre mouths were clearly visible in all of the cut individuals (Figure 6d), except for one of the quarters, and by day 84, 68% of the anemones which were cut in half had mouths that were almost centrally located within the oral disc. This level of regeneration was not found for any of the anemones cut in quarters at any stage of the experiment.

Asexual reproduction did not occur naturally during the experiment; however, sexual reproduction did occur. Eggs were found in two tubs on the 16 January (19 nights after the full moon). One of these anemones had been cut in half, and the other had been cut in quarters 99 d earlier. It is not known if any males spawned, as sperm would have been rapidly removed from the tubs due to the flow-through seawater.

## Discussion

*E. quadricolor* can be propagated asexually by cutting them in half or quarters. The survival rates of the fragments from the sea anemones that were cut in half were greater than has previously been reported (i.e. 89.3 and 93.8% for experiments 1 and 2 respectively vs. 67% in Porat and Chadwick-Furman [22]). Cutting the anemones in quarters produced more individuals; however mortality rates were comparatively higher, the individuals produced were smaller, and also tended to recover and grow more slowly. The latter finding contrasts other growth experiments with intact anemones, where small anemones often tend to grow faster than larger ones [23], [24]. This difference likely arises from the smaller fragments needing more time to heal and grow a mouth. Irrespective of whether anemones are cut in halves or quarters, the methods detailed provide a low technology solution for culturing this species that would allow for the year round production of individuals for the aquarium trade. Furthermore, a major advantage of this technique for industry would be that once procured, desirable colour morphs that attract higher prices could be cloned thereby increasingly profitability.

Feeding increased ODD growth and reduced wet weight loss, whereas it did not significantly influence PDD growth. Ammonia supplements or the presence of anemonefish (which produce wastes that can be used by their host) have similarly been shown to reduce size loss in *E. quadricolor* [25], [26]. It is possible that more frequent feeding may have been necessary to optimise growth during our study. For example, Chomsky et al. [24] found that *Actinia equina* needed to be fed twice a week for significant PDD growth. However, given that the size difference of fed and unfed anemones was greatest in individuals that were not cut, the similarity between feeding treatments in the cut anemones may have been due to the time it took for them to grow a fully functioning mouth (56 d). It is therefore likely that running the experiment for longer would have resulted in a greater feeding effect. Regardless of the treatment, all of the anemones would have received some nutritional benefits from their *Symbiodinium*, microscopic particulate matter, or dissolved organic matter that would have provided energy and potentially influenced size [26]–[28].

Precisely measuring size change in sea anemones using non-destructive methods is not simple given their ability to expand, contract, and store variable amounts of water in the body [29], [30]. In this study, we found that wet weight showed greater variation and less correlation with the linear size measurements of ODD and PDD. Wet weight has been used in a number of studies on sea anemone growth (for example [11], [23], [24]), however these were generally on smaller anemones. Although we did try to standardise the amount of water contained within the anemones it is likely that some variation among individuals was amplified by their larger body size. Despite this, a significant positive influence of feeding on wet weight was still detected.

It is important to recognise that the findings of this study relating to survival might not apply to all sea anemone species. In anemones that use fission as a reproductive mode, wound healing and regeneration occur by rapid cell proliferation at the wound site [31]. Species that do not naturally asexually reproduce may not have this capacity, and therefore may not be able to recover from cutting. Another important ethical consideration is whether cutting the anemones may cause them pain. Cnidarians possess a basic nervous system comprised of a diffuse nerve net with sensory neuron agglomerations at key structures [32]. Column stimulation produces nerve impulses that cause a closure reflex, which might appear to be a nociceptive response; however, nociceptors in Cnidaria only respond to mechanical stimuli and not heat stimuli and without a central processing unit they are unlikely to experience pain [32].

The practical application of the technique developed during this study would allow the creation of a new industry, which could have economic as well as environmental benefits. Collecting pressures on wild populations could therefore be reduced by supplying the aquarium trade with captive-bred anemones. Fragmentation is already used to supply a small percentage of hard and soft corals (which are closely related to sea anemones) for the trade [2], [33], [34]. The study species has been propagated by natural asexual reproduction for research purposes [Chadwick and Delbeek personal communication] and cutting has been used by aquarium hobbyists. Although marine ornamental aquaculture is still in its infancy, it is thought that the propagation of animals for the trade will become more important as further restrictions are placed on wild collection and consumers become more aware of the potential adverse impacts of these activities [12], [13]. The potential for diversification and growth in this sector is promising due to high product value and the benefits to biodiversity conservation; however viability is highly dependent on the price at which individuals can be produced [2], [13].

Because aquarium collecting provides employment in rural low-income coastal areas that have otherwise limited resources and economic options, it would be preferable if aquaculture occurred in the areas that are currently exporting anemones [2], [5], [12]. Given the simplicity of the technique developed during this study it could easily be used without personnel needing a large amount of training, thus allowing livelihoods to be maintained in a more sustainable manner. This may have flow-on benefits such as building the technical capacity of communities and fostering awareness for better stewardship of resources [35]. Furthermore, it may be possible to apply this technique in the field (either in cages or on the reef flat), which would negate the need for captive breeding facilities that are expensive to set up and maintain.

Asexual propagation could also be used for restoration programs and could potentially occur *in situ*. The restocking of areas would help restore the breeding populations needed to help replenish areas that have already been denuded by human or natural disturbances [36]; and therefore have subsequent benefits for anemonefishes that cannot survive in the field without their hosts [10]. Given that this technique would produce individuals with limited genetic variability, the consequences of this would need to be considered [37], along with the causative factors that necessitated restocking in the first place. Management initiatives, such as better regulation and enforcement, may also be needed to address the issues that caused the reductions [36].

The findings of this study demonstrate a feasible solution for reducing aquarium collecting pressures that are impacting host sea anemone abundance in some areas of their distribution [8], [38], and could also allow for the restoration of areas already impacted by human or natural disturbances. Culturing these species would offer economic and environmental benefits by providing an alternative source of these high-value species, which provide essential microhabitat for obligate symbiotic anemonefishes.
